# *In Vitro* Release Kinetics of Antituberculosis Drugs from Nanoparticles Assessed Using a Modified Dissolution Apparatus

**DOI:** 10.1155/2013/136590

**Published:** 2013-07-10

**Authors:** Yuan Gao, Jieyu Zuo, Nadia Bou-Chacra, Terezinha de Jesus Andreoli Pinto, Sophie-Dorothee Clas, Roderick B. Walker, Raimar Löbenberg

**Affiliations:** ^1^Faculty of Pharmacy and Pharmaceutical Sciences, University of Alberta, Edmonton, AB, Canada T6G 2E1; ^2^Faculty of Pharmaceutical Sciences, University of Sao Paulo, 05508-000 Sao Paulo, SP, Brazil; ^3^Merck & Co., Basic Pharmaceutical Sciences, West Point, PA 19486, USA; ^4^Faculty of Pharmacy, Rhodes University, Grahamstown 6140, South Africa

## Abstract

The aim of this study was to assess the *in vitro* release kinetics of antituberculosis drug-loaded nanoparticles (NPs) using a “modified” cylindrical apparatus fitted with a regenerated cellulose membrane attached to a standard dissolution apparatus (modifiedcylinder method). The model drugs that were used were rifampicin (RIF) and moxifloxacin hydrochloride (MX). Gelatin and polybutyl cyanoacrylate (PBCA) NPs were evaluated as the nanocarriers, respectively. The dissolution and release kinetics of the drugs from loaded NPs were studied in different media using the modified cylinder method and dialysis bag technique was used as the control technique. The results showed that use of the modified cylinder method resulted in different release profiles associated with unique release mechanisms for the nanocarrier systems investigated. The modified cylinder method also permitted discrimination between forced and normal *in vitro* release of the model drugs from gelatin NPs in the presence or absence of enzymatic degradation. The use of dialysis bag technique resulted in an inability to differentiate between the mechanisms of drug release from the NPs in these cases. This approach offers an effective tool to investigate *in vitro* release of RIF and MX from NPs, which further indicate that this technique can be used for performance testing of nanosized carrier systems.

## 1. Introduction

Over the past decade, NPs have received significant attention as drug delivery systems, due to significant advantages, including increased drug solubility and bioavailability, reduced toxicity and ability to behave as a drug depot in addition to providing a delivery system using targeting vectors. However, there are no standard methods for the evaluation of the *in vitro* release behaviour of molecules loaded into NPs.

A variety of methods have been reported for *in vitro *drug release evaluation for colloidal drug carriers [1, 2]. Also several methods have been tried to standardize the *in vitro* release test such as a modified USP Apparatus 4 (flow through cell) equipped with a dialysis adapter [3] and a modified USP Apparatus 1 (basket) fixed with a glass cylinder cell to investigate ibuprofen NPs [4]. The data reported for these methods have low standard variations and show different release profiles for different formulations.

**Figure 1:**
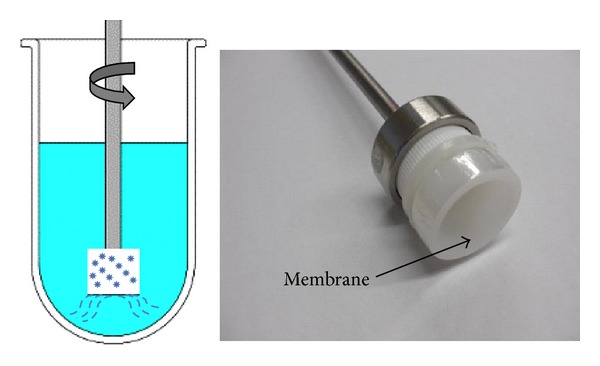
Modified dissolution apparatus for NPs.

**Figure 2:**
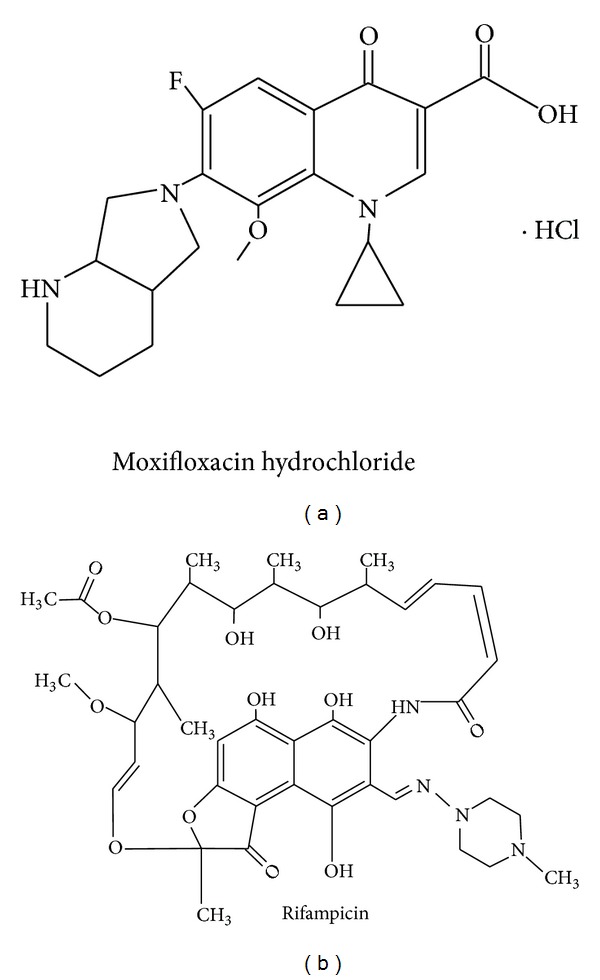
Structures of MX (a) and RIF (b).

In this study, to further investigate the applicability of the modified USP Apparatus 1 that was equipped with a membrane diffusion cylinder, we have assessed *in vitro* release of RIF an MX and the mechanism of drug release from the drug-loaded NPs using this method (Figure 1). Gelatin B and PBCA were selected as the model nanocarriers, respectively. MX is a modified fluoroquinolone antibiotic that is amphoteric with two protonation sites (Figure 2(a)). The pKa values are 6.25 for the carboxylic acid group and 9.29 for the piperazine moiety and it has an isoelectric point of 7.9 [5]. The presence of both a carboxyl and the amine functional group indicates that the molecule will exhibit pH sensitive attributes [6]. RIF is one of the first line antituberculosis agents that also exhibits pKa values at 1.7 (hydroxyl group at position C8) and 7.9 (protonation at the piperazine moiety at position N4). The isoelectric point occurs at a pH of 4.8 [7] (Figure 2(b)). Gelatin B is a protein with both carboxylic and amine functional groups with its isoelectric point between pH 4.6 and pH 5.2. PBCA NPs are the oldest known pharmaceutical NPs [8] and possess a negative charge on their surface. They undergo enzymatic hydrolysis to yield a primary alcohol, butanol, and water-soluble poly(2-cyanoacrylic acid).

The aim of this study was to investigate whether the use of the modified cylinder approach permits discrimination between different *in vitro* release patterns and mechanisms of release of RIF and MX. Drug release was assessed in media of different pH and enzymatic degradation of gelatin NPs was induced with trypsin. The resultant drug release profiles were fitted to the Korsmeyer-Peppas model [9] to establish the predominant drug release mechanisms.

## 2. Materials and Methods

### 2.1. Materials

Gelatin B (225 Bloom), glutaraldehyde (25% w/w aqueous solution), and trypsin were obtained from Sigma-Aldrich (Ontario, Canada). N-butyl cyanoacrylate monomer was a gift from Loctite Ltd. (Dublin, Ireland). RIF was obtained from PCCA (Ontario, Canada) and MX from Wanquan Pharmaceuticals (Beijing, China). Dialysis membranes were from Spectrum Laboratories Inc. (Rancho Domi, guez, CA, USA). All chemicals were of analytical grade.

### 2.2. Preparation of Drug-Loaded Gelatin NPs

MX loaded gelatin NPs (MX-Gel-NPs) and RIF loaded gelatin NPs (RIF-Gel-NPs) were manufactured following a two-step desolvation process as has been previously described [10]. In brief, 1.25 g gelatin was dissolved in 25 mL double distilled water (ddH_2_O) under constant heating in the temperature range 30–40°C. A 25 mL aliquot of acetone was added to the gelatin solution as a desolvating agent to precipitate the gelatin. The supernatant was discarded and the gelatin was redissolved by adding 25 mL ddH_2_O and stirring at 600 rpm under constant heating. The pH of the gelatin solution was adjusted to 2.5. Acetone (75 mL) was added dropwise to facilitate the formation of NPs. Approximately 10 mg of MX or 5 mg RIF was dissolved in acetone at concentration of 1 mg/mL or 2 mg/mL, respectively, and was added to the NPs after 1 h. At the end of the process, 250 *μ*L of 25% w/w glutaraldehyde solution was added to the solution as a cross-linking agent, and the mixture was stirred for 12 h at 600 rpm. Acetone was removed by evaporation using a rotary evaporator (IKA, Staufen, Germany). The resultant NPs were purified by centrifugation at 8000 rpm for 30 min (Beckman L8-M ultracentrifuge, CA, USA) and washed three times with ddH_2_O. The NPs were collected and filtered through a hydrophilic 0.45 *μ*m polyvinylidene fluoride filter (Millipore, Billerica, MA, USA), followed by lyophilization for 24 h at −50°C and 45 Pa.

### 2.3. Preparation of Drug-Loaded PBCA NPs

MX loaded PBCA NPs (MX-PBCA-NPs) and RIF loaded PBCA NPs (RIF-PBCA-NPs) were manufactured by anionic polymerization as previously described [11]. Briefly, a 1% v/v solution of n-butyl-2-cyanoacrylate was added dropwise to a 1% m/v dextran in 0.01 N HCl solution with constant stirring at 600 rpm for 30 min after which drug was added to the mixture. After 3 h of exposure the reaction was stopped by neutralization with 0.1 N NaOH. The particles were purified by centrifugation at 8000 rpm for 30 min and washed three times with ddH_2_O. The NPs were collected and filtered through a 0.45 *μ*m filter prior to lyophilization at −50°C and 45 Pa and further studies.

### 2.4. Drug Loading

MX-Gel-NPs or RIF-Gel-NPs powders were dispersed in 5 mL of a trypsin solution (0.2 mg/mL) in a 10 mL flask and shaken until a clear colorless solution formed, indicating that complete digestion of the gelatin NPs and release of all MX or RIF encapsulated in the matrices of the NPs had been achieved. Methanol was added to the flask and the solution was made up to volume, filtered through a 0.45 *μ*m filter and analyzed using a validated HPLC method.

For the PBCA NPs, the drug loading was calculated as the difference between the initial drug concentration and the drug concentration found in the supernatant of unwashed NPs suspension using HPLC [12].

The drug loading of the MX-Gel-NPs, MX-PBCA-NPs and RIF-PBCA-NPs was 6.622 ± 0.1124% w/w, 50.41 ± 2.323% w/w, and 5.157 ± 1.231% w/w, respectively, and that of the RIF-Gel-NPs was 21.60 ± 1.861% w/w and 56.71 ± 1.280% w/w.

### 2.5. *In Vitro* Drug Release Using the Modified Cylinder Method and Dialysis Bag Technique

Two methods based on membrane separation techniques were used to evaluate the *in vitro* release of RIF and MX from the NPs formulations. One approach was the use of the modified cylinder method and the other the use of dialysis.

The modified cylinder method required that 5 mg of NPs were suspended in 2 mL of release media and placed into a flat-bottom cell (internal diameter 2 cm) with the opening covered using dialysis membrane (MWCO: 12–14 kDa). The *in vitro *release study was performed using USP dissolution Apparatus 1 by fixing the modified cylinder onto a basket shaft and operating the apparatus at 100 rpm. A 100 mL aliquot of release media was used at 37°C. At designated time intervals, 1 mL of samples were collected and the withdrawn media was replaced with fresh media. The release media were phosphate buffer solution (PBS) of pH 7.4, acetate buffer of pH 4.0, and HCl buffer of pH 1.2, respectively. The drug concentrations were measured using a validated HPLC assay.

The dialysis bag technique entails dispersing 5 mg of the NPs in 2 mL of the release medium and placing in a dialysis bag (MWCO: 12–14 kDa, surface area of 22.5 cm^2^), which was then submerged in a conical flask that contained 100 mL of the test media maintained at 37°C and that was stirred at 100 rpm. At designated time intervals, 1 mL aliquots were collected and replaced with fresh media. The drug concentrations were determined using a validated HPLC assay.

### 2.6. Drug Diffusion Behaviour through the Dialysis Membrane

Solutions of MX and RIF as free drug were prepared in different release media of pH 1.2, 4.0, and 7.4. Solutions of drug containing an equivalent dose to that in the NPs (2.6 mg MX, 1.1 mg RIF, or 2.9 mg RIF) were placed in the modified cylinder apparatus. The dialysis membranes of MWCO 12–14, 25, and 50 kDa have been used for the experiment. The diffusion experiments were performed in 100 mL of the specific release media maintained at 37°C and agitated at 100 rpm. At predetermined time intervals, 1 mL samples were withdrawn and analyzed using a validated HPLC assay to determine the amount of drug that had been released.

### 2.7. Kinetic Analysis of Drug Release Profiles

The drug release data were computed using DDsolver, which is an Excel-plugin module [13] and the resultant data were fitted to the Korsmeyer-Peppas exponential equation (1) to establish the mechanism of drug release

(1) $$\begin{array}{c} Q=kt^{n}, \end{array}$$

where *Q* is the percentage of drug released at time *t* and *k* is a constant incorporating the structural and geometric characteristics of the device under investigation. The diffusional exponent *n* is an important indicator of the mechanism of drug transport from the dosage form. A value of *n* ≤ 0.43 indicates that drug release is controlled by Fickian diffusion, whereas a value of *n* ≥ 0.85 suggests that drug release is dominated by an erosion mechanism. For values 0.43 < *n* < 0.85, the release is described as anomalous, implying that a combination of diffusion and erosion contributes to the control of drug release.

### 2.8. HPLC Analysis

The concentration of MX and RIF was determined by reversed-phase HPLC using a LiChrocart-LiCrospher 100 RP-18, 5 *μ*m stationary phase (Merck, Darmstadt, Germany). The mobile phase consisted of a mixture of methanol and 0.3% v/v triethylamine-0.02 M PBS (pH 3.0) (40 : 60 v/v) for the analysis of MX and a 20 *μ*L sample was injected at a flow rate of 0.9 mL/min with UV detection at 295 nm as reported [14]. In 0.1 N HCl buffer with pH 1.2, acetate buffer with pH 4.0, and PBS with pH 7.4, the linear regression equations obtained were *A* = 94.48*C* + 31.29 (*r* ^2^ = 0.9996, *n* = 5), *A* = 90.31*C* − 62.53 (*r* ^2^ = 0.9994, *n* = 5), and *y* = 84.69*C* − 16.35 (*r* ^2^ = 0.9997, *n* = 5), respectively. *A* is the absorbance and *C* (*μ*g/mL) is the concentration.

The mobile phase used for the analysis of RIF comprised of a mixture of methanol and 10 mM ammonium acetate (60 : 40 v/v) as previously reported [15]. The RIF samples were analyzed at a flow rate of 0.9 mL/min with UV detection at 337 nm. The linear regression equation was *A* = 28.25*C* + 15.17 (*r* ^2^ = 0.9990, *n* = 5).

## 3. Results and Discussion

### 3.1. Diffusion Rate of Free Drug Solutions Using the Modified Cylinder Method

The advantage of using dialysis membranes is that they can be used to separate the dialyzed solution containing drug from NPs matrices. However dialysis membranes may limit drug release [16, 17] and therefore, to prevent the impact of the membrane on drug diffusion, a large pore size dialysis membrane should be selected. Thus, the diffusion of MX and RIF from solution through the membrane with MWCO of 12–14, 25, and 50 kDa in different media was tested. As shown in Table 1, only a very slightly but not significantly increase of t50% was observed for MX and RIF upon increasing the membrane pore size (*P* > 0.01). When membrane of MWCO of 12–14 kDa was used, t50% of the free MX solution in different release media of pH 1.2, 4.0, and 7.4 was achieved within 7–9 min. t50% values of free RIF solution in media of pH 7.4 and 4.0 were within 13–16 min. The results confirm that dissolved drug molecules readily pass freely through the dialysis membrane. This may be because the molecular weight of the MX and RIF was much smaller than the pore sizes of membrane [4, 16]. Accordingly, MWCO of 12–14 kDa membranes were selected for all future release tests. In addition, the maximum concentration in the dialysate of free RIF solution in medium of pH 1.2 reached 52.82% at 2 h, which may be due to the formation of an ion pair between RIF and Cl^−^ when the pH is ≤2.6 [18]. Another reason for this observation may be due to degradation of RIF in acid [19, 20]. Similar results were reported by Abdel-Mottaleb and Lamprecht [4].

### 3.2. *In Vitro* Release of MX from MX-Gel-NPs

**Figure 3:**
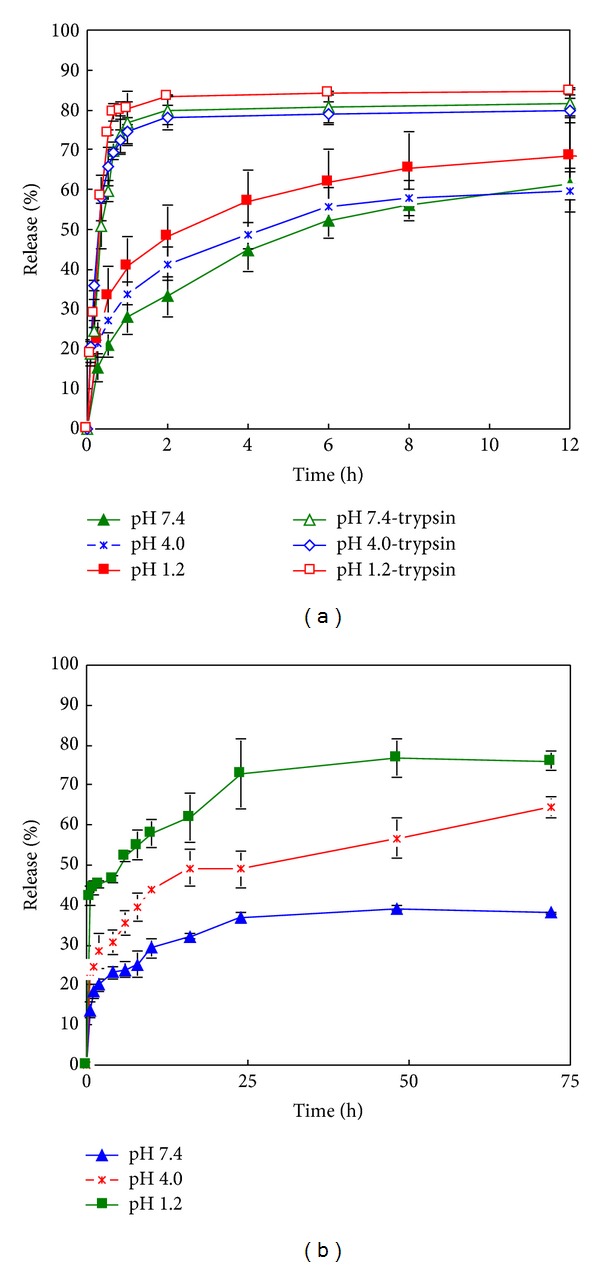
*In vitro* release curves of MX-NPs in media with different pH using the modified cylinder method. (a) MX-Gel-NPs in the presence and absence of trypsin; (b) MX-PBCA-NPs. Data shown is the mean ± S.D. (*n* = 4).

The release of MX from MX-Gel-NPs was performed in buffers of different pH in the presence or absence of the enzyme, trypsin at 37°C. The MX release profiles over time are shown in Figure 3(a) at different pH values in the absence of trypsin. Approximately 26–44% MX is released within 1 h. In contrast, the release of MX in media containing 0.05% w/w trypsin increases significantly (*P* < 0.05), with more than 80% of MX releases after 1 h in all media. The forced degradation of NPs using digestive enzymes produces a significant increase in the extent of MX released in both media. In addition, as shown in Figure 3(a), the drug release tends to be lower at high pH values. This may in part be due to the lower solubility of MX at this pH. Langlois et al. reported that MX is most lipophilic at pH 7.4 due to the presence of neutral and zwitterions forms, which may explain the slow dissolution rate observed [5].

### 3.3. *In Vitro* Release of MX from MX-PBCA-NPs

All *in vitro* release profiles from MX-PBCA-NPs show an initial burst release within the first 10 minutes (Figure 3(b)), which may be associated with the distribution of MX on the surface of PBCA NPs. The location of the drug on the surface of the particles permits instantaneous dissolution when it comes in contact with the dissolution medium. Surface adsorption seems to be a predominant interaction between this fraction of MX and surface of PBCA particles. The remaining fraction is located inside the NPs matrix and is released slowly which supports reports that PBCA NPs exhibit a biphasic release pattern with an initial burst effect followed by a sustained release of the drug contained in the particle [21].

### 3.4. *In Vitro* Release of RIF from RIF-Gel-NPs

**Figure 4:**
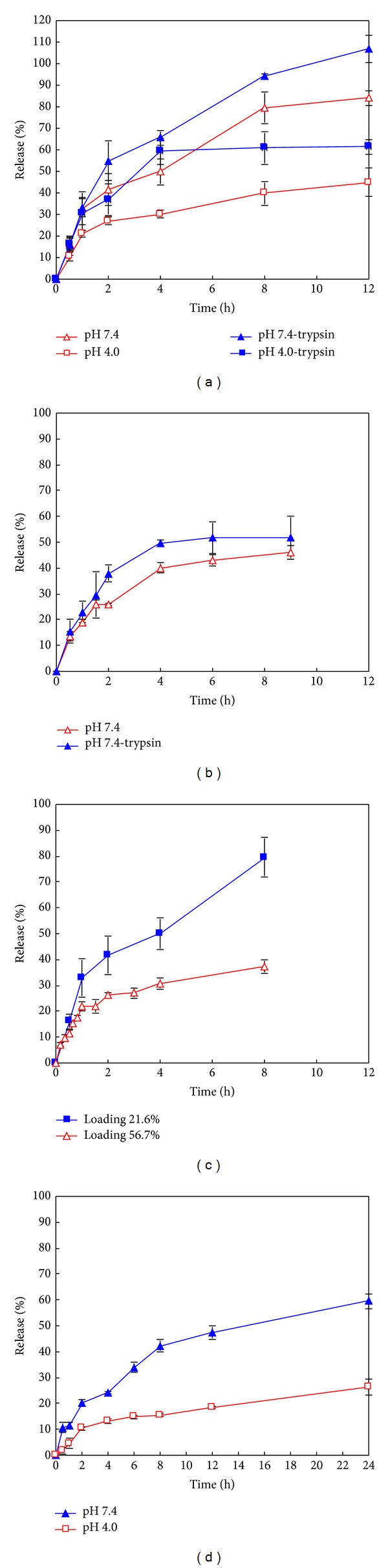
*In vitro* release curves of RIF-NPs using the modified cylinder method. (a) RIF-Gel-NPs with drug loading of 21.6% w/w in the presence and absence of trypsin; (b) RIF-Gel-NPs with drug loading of 56.7% w/w in the presence and absence of trypsin; (c) RIF-Gel-NPs with different loadings in PBS with pH 7.4; (d) RIF-PBCA-NPs in buffers with different pH values. Data shown is the mean ± S.D. (*n* = 3).

The *in vitro* release profiles of RIF from RIF-Gel-NPs over time in different media are shown in Figures 4(a) and 4(b). In the absence of trypsin, RIF is released extremely slowly when compared to the release profiles observed when digestive enzymes are present in the dissolution medium. It is also established that the release of RIF is also dependent on drug loading (Figure 4(c)). A higher drug loading results in a slower rate of drug release. In addition RIF release is found to be pH dependent. As shown in Figure 4(a), the higher extent of drug release is observed in buffer of pH 7.4 with a lower amount of drug release occurring in a medium of pH 4.0. The concentration of RIF in medium of pH 1.2 in which trypsin is omitted is below the limit of detection and approximately 1–5% RIF is released from RIF-Gel-NPs when trypsin is present (Data not shown), which is similar to the results reported by Bhise and Mookkan [22].

The loading efficiency of NPs is dependent on the properties of the polymer used and the physicochemical characteristics of the drug to be incorporated into the particles. The interaction of RIF with gelatin NPs is dependent on three factors, namely, hydrogen bonding, electrostatic interaction, and hydrophobic forces [23, 24]. Firstly, hydrogen bonding occurs in the presence of hydroxyl functional groups of the RIF molecule and carboxyl groups of gelatin B. Secondly, there are electrostatic interactions between molecules (namely, molecules of the gelatin B and molecules of RIF.) Gelatin exhibits a net negative charge at a pH of 7.4 due to the predominance of negative –COO^−^ functional groups which repel the partly anionic RIF at this pH [25]. Therefore electrostatic repulsion may facilitate a greater extent of drug release in these solutions. Similar results were reported by Bajpai and Choubey [26]. The greater solubility of RIF at pH 7.4 than that at pH 4.0 may also explain the higher dissolution rate observed from RIF-Gel-NPs at this pH. Due to the fact that the molecule exhibits two pKa values, a biphasic solubility curve is expected. The solubility of RIF has been reported as 125–127.2 mg/mL at pH 1.0–1.4 [27], 3.35 mg/mL at pH 7.4, and 0.99 mg/mL at pH 4.0 [28]. However, the slowest rate of RIF release is observed at a pH of 1.2 despite its relatively high solubility at this pH. Part of the reason for this observation may be due to degradation of RIF in acid. Another possible explanation for this behaviour may be that RIF and Cl^−^ form an ion pair when the pH is ≤2.6 [18]. This effect seems to play a significant role in respect of drug release in a solution of pH 1.2. Finally, hydrophobic forces might also impact drug release and it has been reported that hydrophobic forces were responsible for binding of RIF to bovine serum albumin [29]. In our study, the high drug loading w/w is accompanied with slow drug release and this might be due to the presence of hydrophobic interactions between RIF and gelatin.

### 3.5. *In Vitro* Release of RIF from RIF-PBCA-NPs

The release profiles of RIF released from RIF-PBCA-NPs are shown in Figure 4(d). The rate of drug release is slower when compared to that observed for the gelatin NPs and this may be due to the hydrophobic interactions of RIF with the PBCA matrix leading to a slower and lower extent of drug release.

The pH-dependent release of RIF from RIF-PBCA-NPs can be explained by a similar mechanism as that proposed for RIF release from RIF-Gel-NPs. At pH 1.2, RIF is able to form an ion pair with Cl^−^ ions, which in turn decreases the extent of drug release from PBCA NPs. The higher solubility observed at pH 7.4 compared to pH 4.0 contributes a higher drug release of RIF at this pH. In addition, the zwitterionic RIF molecule (~40% anionic) is repelled from the anionic PBCA matrix, which increases the drug release rate at pH 7.4.

### 3.6. Comparison of Dialysis Bag Technique for Release from MX-Gel-NPs and RIF-Gel-NPs

**Figure 5:**
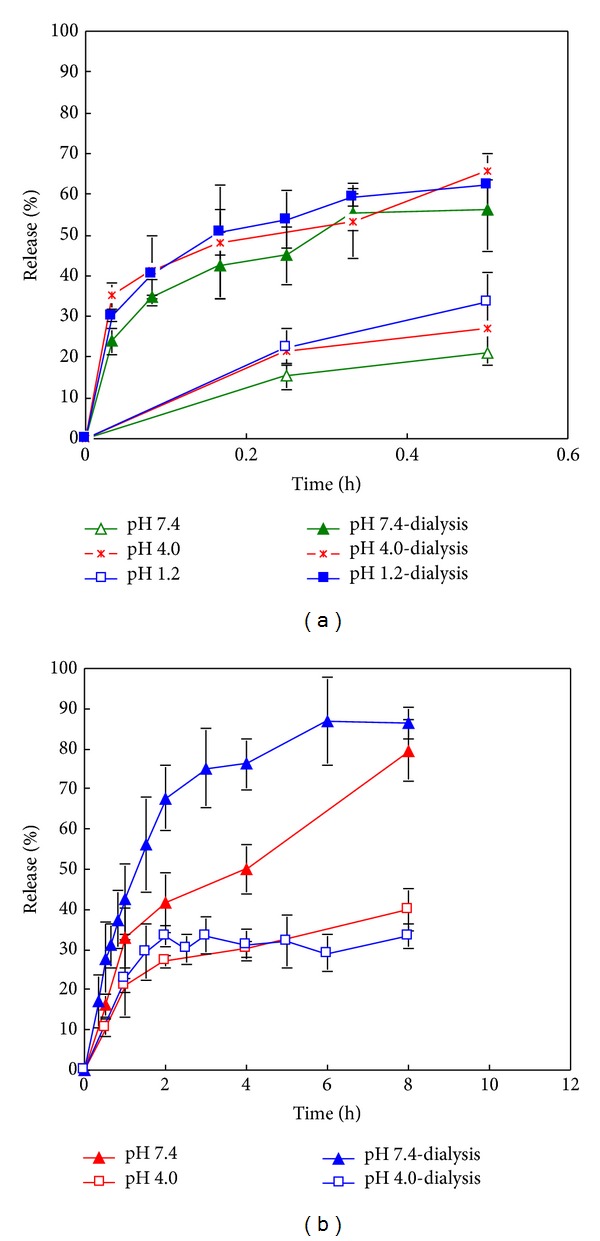
*In vitro* release curves of gelatin NPs using the modified cylinder method and the dialysis bag technique. (a) MX-Gel-NPs; (b) RIF-Gel-NPs with drug loading of 21.6% w/w.

**Figure 6:**
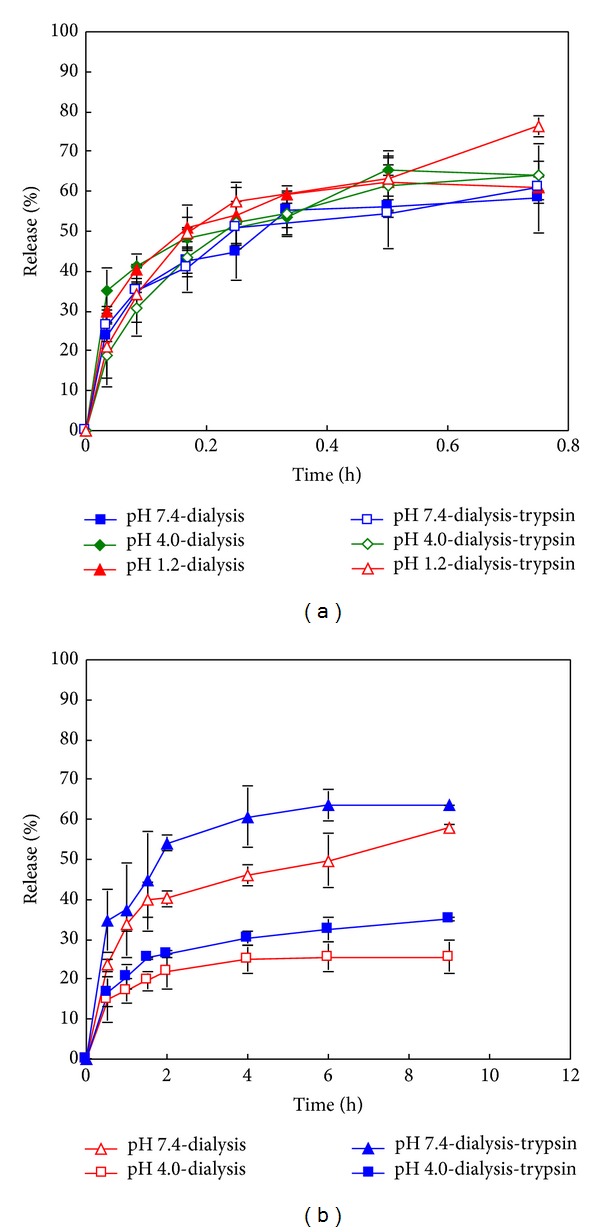
*In vitro* release curves of gelatin NPs in the presence and absence of trypsin. (a) MX-Gel-NPs using dialysis bag; (b) RIF-Gel-NPs with drug loading of 56.7% w/w. Data shown is the mean ± S.D. (*n* = 3).

The pH-dependent dissolution behaviours of MX and RIF are similar using both methods of drug release that were investigated (Figures 5(a) and 5(b)). However when the modified cylinder method was used, a much slower drug and no burst release was observed. This might be caused by the relatively smaller release area compared to dialysis bags. Similar results were reported by Abdel-Mottaleb and Lamprecht [4]. The release profiles of MX-Gel-NPs (Figure 6(a)) and RIF-Gel-NPs (Figure 6(b)) in buffers with or without trypsin using the dialysis bag technique are shown in Figure 6. It is clear that the use of the dialysis bag technique does not permit differentiation between studies in which trypsin was included (forced) in the dissolution medium or not (nonforced) for MX-Gel-NPs.

### 3.7. Kinetic Assessment and Release Mechanisms

The regression coefficients (*r*-values) generated following fitting of drug release data to the Korsmeyer-Peppas model equation are summarized in Tables 2–4 for all formulations tested. As observed in Table 2, the release data of MX-Gel-NPs without trypsin using the modified cylinder method is fitted to the Korsmeyer-Peppas equation with a Fickian release exponent (*n* = 0.2538–0.3640), suggesting that the drug release from the NPs occurs primarily via diffusion. The significant MX release observed in the presence of trypsin at pH 4.0 and pH 7.4 is best described by an anomalous transport mechanism (*n* = 0.6910–0.6918) and the release at pH 1.2 is described by a Case II transport process (*n* = 0.8966). This transport is characterized by polymer relaxation due to polymer erosion when enzymatic degradation occurs [30].

The release data observed for the RIF-Gel-NPs manufactured using different drug loading fit well to the Korsmeyer-Peppas equation with an *n* value from 0.3543 to 0.4254, which suggest that a Fickian diffusion process is predominant (Table 3). In the presence of trypsin, RIF release from RIF-Gel-NPs is best described as anomalous transport (*n* = 0.5432–0.5728) due to the erosion of the gelatin matrix by enzymatic degradation.

From above, the use of the modified cylinder method permits differentiation between the presence and absence of trypsin in the cases of the MX-Gel-NPs (Table 2) and RIF-Gel-NPs (Table 3). However, the use of the dialysis bag technique results in poor differentiation between forced and nonforced drug release studies in all the cases. The release of MX from gelatin NPs using the dialysis bag is best fitted to a Fickian diffusion model in both cases (*n* = 0.1899–0.3902) (Table 2) and there is no obvious difference in the release profiles in the presence or absence of trypsin (Figure 6(a)). Though the release profiles of RIF from gelatin NPs with high drug loading using dialysis bag can be differentiated in the presence or absence of trypsin for RIF-Gel-NPs (Figure 6(b)), their kinetics mechanism can only be described by Fickian diffusion (*n* = 0.2555–0.2994) in both cases (Table 3).

The release mechanisms of MX-PBCA-NPs and RIF-PBCA-NPs are summarized in Table 4. The release of MX-PBCA-NPs is found to be Fickian diffusion controlled (*n* = 0.1054–0.2256) for the first 10 mins after which sustained release is observed. Furthermore, the mechanism of RIF release from RIF-PBCA-NPs is best described by an anomalous transport mechanism (*n* = 0.4531–0.4612). This is different to the release observed for the RIF-Gel-NPs which exhibit Fickian diffusion. The different release mechanism of RIF from gelatin NPs and PBCA NPs may be due to hydrophobic interactions between the two nanocarrier matrices and RIF.

### 3.8. Advantages of the Modified Cylinder Method

To evaluate the *in vitro* release mechanisms of pharmaceutical dosage forms, a dissolution system should operate under sink conditions and be able to discriminate release characteristics that may be a consequence of variation in formulation composition. Ideally the *in vitro* release profiles should mimic the *in vivo *release mechanisms as much as possible. The modified cylinder method described and used in our study is able to discriminate and facilitate the elucidation of the *in vitro* release mechanism of drug from NPs formulation in different media, whereas the dialysis bag technique is not useful in this respect in these cases. In addition, the standard deviation of the release data using the modified cylinder method is tighter compared to that observed for the dialysis bag technique (Figures 5(a) and 5(b)). These findings may be attributed to the constant surface area as well as constant hydrodynamic conditions that exist at the surface of the membrane used in the modified cylinder apparatus. The hydrodynamic conditions at the surface of dialysis bags might vary slightly when the bags are stirred and use of the modified cylinder in standard dissolution equipment may also be an advantage.

## 4. Conclusions

The drug release profiles observed for the different NP formulations manufactured in these studies exhibited different mechanisms of release. The dialysis bag technique was compared to a modified cylinder method using a standard dissolution apparatus. The use of the modified cylinder method facilitated identification of the release kinetics of the two model drugs depending on their pH-dependent solubility, electrostatic interaction with the nanocarriers, and the impact of hydrophobic forces. In addition the impact of enzymatic degradation on drug release was readily observed. Use of the dialysis bag technique did not permit differentiation between forced and nonforced drug release from gelatin NPs in these cases. Though the modified cylinder method has not yet been used to mimic* in vivo* conditions, it offers an alternate evaluation tool to investigate *in vitro *drug release from NPs. This approach and apparatus can be considered and used as a performance test for quality control testing of nanosized delivery systems. The utility of this new method to establish *in vitro/in vivo* relationships needs to be further investigated.

## Figures and Tables

**Table 1:** Comparison of t50% for free drug diffusion through dialysis membranes with different MWCO in media.

Free drug/t50%	MWCO of membrane (kDa)		
	12–14	25	50
MX	7–9 min	5–7 min	5–7 min
RIF	13–16 min	12–14 min	11–13 min

**Table 2:** Kinetic assessment of release data of MX-Gel-NPs in diverse buffers (*Q* < 0.6).

Methods	Media	Korsmeyer-Peppas	
		*n* ^a^	*R* ^2^ ^b^
Modified cylinder method	pH 7.4	0.3640	0.9956
	pH 4.0	0.2538	0.9765
	pH 1.2	0.2944	0.9631
	pH 7.4-trypsin	0.6918	0.9503
	pH 4.0-trypsin	0.6910	0.9979
	pH 1.2-trypsin	0.8966	0.9780

Dialysis bag technique	pH 7.4	0.3032	0.9875
	pH 4.0	0.1899	0.9893
	pH 1.2	0.2891	0.9863
	pH 7.4-trypsin	0.3902	0.9825
	pH 4.0-trypsin	0.2678	0.9824
	pH 1.2-trypsin	0.3614	0.9122

^a^Diffusional exponent and ^b^squared correlation coefficient.

**Table 3:** Kinetic assessment of release data of RIF-Gel-NPs in diverse buffers (*Q* < 0.6).

Method	Drug loading (w/w)	Media	Korsmeyer-Peppas	
			*n* ^a^	*R* ^2^ ^b^
Modified cylinder method	21.6%	pH 7.4	0.4254	0.9541
		pH 4.0	0.3543	0.9478
		pH 7.4-trypsin	0.5501	0.8854
		pH 4.0-trypsin	0.5728	0.9636
	56.7%	pH 7.4	0.3911	0.9245
		pH 7.4-trypsin	0.5432	0.9774

Dialysis bag technique	21.6%	pH 7.4	0.6764	0.9721
		pH 4.0	0.5472	0.9760
	56.7%	pH 7.4	0.2779	0.9397
		pH 4.0	0.2555	0.9842
		pH 7.4-trypsin	0.2994	0.9098
		pH 4.0-trypsin	0.2744	0.9772

^a^Diffusional exponent and ^b^squared correlation coefficient.

**Table 4:** Kinetic assessment of release data of MX-PBCA-NPs and RIF-PBCA-NPs in diverse buffers (*Q* < 0.6).

Model drug	Media	Korsmeyer-Peppas	
		*n* ^a^	*R* ^2^ ^b^
MX	pH 7.4	0.2256	0.9697
	pH 4.0	0.2165	0.9653
	pH 1.2	0.1054	0.8442

RIF	pH 7.4	0.4531	0.9762
	pH 4.0	0.4612	0.9510

^a^Diffusional exponent and ^b^squared correlation coefficient.
